# Exploring the taxonomical and functional profiles of marine microorganisms in Submarine Groundwater Discharge vent water from Mabini, Batangas, Philippines through metagenome-assembled genomes

**DOI:** 10.3389/fgene.2025.1522253

**Published:** 2025-02-10

**Authors:** Joshua T. Veluz, Laurence Anthony N. Mallari, Paul Christian T. Gloria, Maria Auxilia T. Siringan

**Affiliations:** ^1^ Natural Sciences Research Institute, College of Science, University of the Philippines Diliman, Quezon City, Philippines; ^2^ Institute of Biology, College of Science, University of the Philippines Diliman, Quezon City, Philippines

**Keywords:** biosynthethic gene clusters, metagenome-assembled genomes, nutrient metabolism genes, shotgun sequencing, submarine groundwater discharge, vent water

## 1 Introduction

Submarine groundwater discharge (SGD) refers to the movement of water from land to coastal waters, flowing across the land-ocean interface (Adyasari et al., 2019). SGD is ubiquitous in sandy, rocky, and muddy shorelines and may include fresh groundwater of terrestrial origin, recirculated seawater, or a combination of both (Adyasari et al., 2019; Santos et al., 2021). The presence of SGD in these areas results in physical and chemical gradients that create unique biogeochemical environments. SGD acts as a conduit for the transport of materials such as gases, nutrients, and trace metals, from land to sea (Moore, 2010; Knee and Paytan, 2011). The flux of nitrogen and phosphorus to the ocean from total SGD, which includes both fresh and recirculated seawater, is estimated to exceed riverine inputs on a global scale (Cho et al., 2018). The SGD-mediated inflow of nutrients can significantly impact coastal ecosystems and water quality, altering levels of dissolved and gaseous metabolites, including ammonium, methane, and hydrogen sulfide (Bernard et al., 2014; Santos et al., 2021; Schlüter et al., 2004). This influences microbial communities and their metabolic activities in these specific locations (Purkamo et al., 2022).

Similar to terrestrial subsurface environments, deep marine sediments are also characterized by an absence of photosynthetically produced labile organic carbon (Chen et al., 2023). Because of this, groundwater microorganisms have developed diverse strategies to ensure survival and persistence. Among these strategies is the ability to utilize ancient organic carbon from rocks, allochthonous organic carbon, or byproducts from the degradation of organic contaminants (Griebler and Lueders, 2009; Smith et al., 2015). Other groundwater microorganisms also have adaptations that enable them to fix inorganic carbon by utilizing energy from the oxidation of substrates such as nitrite, ammonium, reduced iron, and sulfur compounds (Ruiz-González et al., 2021).

Applying shotgun metagenomics and assembling microbial genomes from metagenomic data can reveal important insights into the structure-function relationships of microbial communities within complex environments (Overholt et al., 2020). Through the use of metagenome-assembled genomes (MAGs), researchers can reconstruct near-complete microbial genomes, enabling precise taxonomic identification and comprehensive functional profiling of these assembled genomes (Mangoma et al., 2024).

For instance, Mangoma et al. (2024) successfully recovered taxonomically diverse MAGs from Buhera soda pans in Zimbabwe. These genomes revealed metabolic pathways associated with nitrogen fixation and sulfur cycling, highlighting the ecological roles of microbial communities in such extreme environments. Similarly, a study by He et al. (2016) leveraged metagenomic sequencing of sediment samples from deep-sea hydrothermal vents in the Guaymas Basin, Mexico, to identify *Bathyarchaeota* MAGs. These genomes were found to contain genes encoding the Wood-Ljungdahl (WL) pathway, an acetogenic process crucial for carbon fixation and acetate production. This finding underscores the pivotal role of *Bathyarchaeota* in the carbon cycle within deep-sea vent ecosystems.

Building on the work of Mangoma et al. (2024) and He et al. (2016), this data report seeks to deepen our understanding of specific environments, such as SGDs, from a microbial perspective. This report expands our knowledge of microbial species thriving in such environments, sheds light on their functional roles, and reveals untapped resources for industrial and medical applications through the analysis of these assembled genomes.

In this study, high-quality MAGs were generated from shotgun metagenome data derived from water samples from an SGD vent in Mabini, Batangas, Philippines. The presence of SGD in the collection site was documented by Cardenas et al. (2020) using Radon (^222^Rn) concentrations, a natural tracer for SGD (Burnett et al., 2003; Burnett et al., 2006; Cardenas et al., 2020). The generated MAGs were annotated to identify their potential environmental roles and subsequently compared to existing genomes for further insights. This data report offers preliminary findings that can serve as a foundation for future studies. It provides valuable insights into the limited research on the microbial dimension in SGD-associated areas within the Philippines.

## 2 Methods

Water samples (4 L) were collected from an SGD vent in the Sea Spring site in a coastal area in Mabini, Batangas, Philippines (13.68701 N, 120.89573 E). Divers brought a 4 L-Nalgene bottle filled with surface water to the floor of the Sea Spring site, about 7 m in depth. The surface water was purged from the bottle using pressurized air from the divers’ tanks. After purging, the bottles were titled towards the vent to collect the SGD water. Using a vacuum pump filtration system, the water samples were filtered through a 3 µm polycarbonate track-etched (PCTE) membrane (Sterlitech Corp., United States). After filtration, the membrane filter was placed in a cryovial and stored in a portable cryotank containing liquid nitrogen before transporting to the laboratory.

Total DNA was extracted from the membrane filter using the DNeasy PowerSoil^®^ Pro Kit (QIAGEN), following the manufacturer’s protocol with minimal modifications. Specifically, three sample replicates were prepared, each subjected to the lysis step, and combined in a single column to ensure sufficient DNA concentration. The concentration of the extracted DNA was determined using the Denovix QFX Fluorometer and dsDNA broad-range kit following the manufacturer’s instructions. The DNA extract was sent to Macrogen, Inc. (South Korea) for shotgun metagenomic sequencing, at 25 Gb throughput, and 150 bp paired-end setting using the Illumina NovaSeq™ 6,000 system.

*De novo* metagenome assembly was performed using various bioinformatics tools in the KBase v2.7.11 platform (Arkin et al., 2018). After merging the forward and reverse reads during the importing stage, the resulting merged raw reads were analyzed with FastQC v0.12.1 (Andrews, 2010) and yielded 77,717,154 total sequences, 11.7 Gbp total bases, 45% G+C content, and no poor-quality sequences. Consequently, the merged raw reads were trimmed using Trimmomatic v0.36 (Bolger et al., 2014) with a sliding window size of 4 and a minimum quality set to 30. The Q30-trimmed reads were assembled using three different assemblers namely: metaSPAdes v3.15.3 (Nurk et al., 2017; Prjibelski et al., 2020), MEGAHIT v1.2.9 (Li et al., 2016), and IDBA-UD v1.1.3 (Peng et al., 2012), all using default parameters. The assemblers metaSPAdes, MEGAHIT, and IDBA-UD showed a total DNA length of 187,749,451 bp, 178,126,656 bp, and 108,098,378 bp, respectively. The metaSPAdes assembly, which had the longest total sequence length, was selected for downstream processing. The metagenomic contigs from the assembly chosen were grouped into bins using three binning tools–CONCOCT v1.1 (Alneberg et al., 2014), MaxBin2 v2.2.4 (Wu et al., 2015), and MetaBAT2 v1.7 (Kang et al., 2019), following default parameters (minimum contig length of 300 ≤ 2000). The generated bins from these three binning tools were consolidated and optimized using the DAS Tool v1.1.2 (Sieber et al., 2018) using default parameters (diamond, 0.5 score threshold, 0.6 duplicate penalty, and 0.5 megabin penalty). The generated bins from DAS Tool optimization were quality-filtered using CheckM v1.0.18 (Parks et al., 2015) to have ≥90% completeness and ≤5% contamination according to the high-quality MiMAG standards (Bowers et al., 2017). The completeness of the genomes was also determined using BUSCO v5.4.6 (Simão et al., 2015). MAGs’ information and taxonomic identities were visualized using the *circlize* package (Gu et al., 2014) in RStudio (RStudio Team, 2024).

The filtered bins were compared to the top 10 closest known genomes using the SpeciesTreeBuilder v0.1.4 (Arkin et al., 2018), which analyzes 49 clusters of orthologous groups (COGs) to determine phylogenetic relationships. In this report, tree reconstruction was conducted without an outgroup to maintain focus on the relationships among the GenBank genomes, allowing for a more refined understanding of their phylogenetic proximity. Consequently, all genomes underwent general annotation using Rapid Annotation using Subsystem Technology (RAST) through the SEED viewer v2.0 platform (Aziz et al., 2008; Overbeek et al., 2013) using the RASTtk annotation scheme. This report specifically highlights genes with critical functions in biogeochemical cycles focusing on those involved in sulfur metabolism, iron acquisition and metabolism, potassium metabolism, phosphorus metabolism, and nitrogen metabolism. The phylogenetic trees and RAST results were visualized using *ggtree* (v1.14.6; Yu et al., 2016) and *ggplot* (v3.5.1; Wickham, 2016) packages through RStudio (RStudio Team, 2024). The bins were also subjected to Distilled and Refined Annotation of Metabolism (DRAM) v0.1.2 (Shaffer et al., 2020) using default settings. To determine the functional COGs, eggNOG-mapper v2 (Cantalapiedra et al., 2021) with eggNOG v5.0 (Huerta-Cepas et al., 2018) were utilized using default settings. Afterward, the resulting .csv files were manipulated in RStudio (RStudio Team, 2024) using the packages *dplyr* (v1.1.4; Wickham et al., 2023), *readr* (v2.1.5; Wickham et al., 2024), and *stringr* (v1.5.1; Wickham, 2023) to generate relative frequencies of COG categories for each MAG and across all MAGs.

Lastly, genome mining of biosynthetic gene clusters (BGCs) was also performed using antiSMASH v7.0 (Blin et al., 2023) with a relaxed detection strictness. The predicted BGCs from the seven high-quality MAGs were recorded and tallied.

## 3 Data and analysis

**FIGURE 1:**
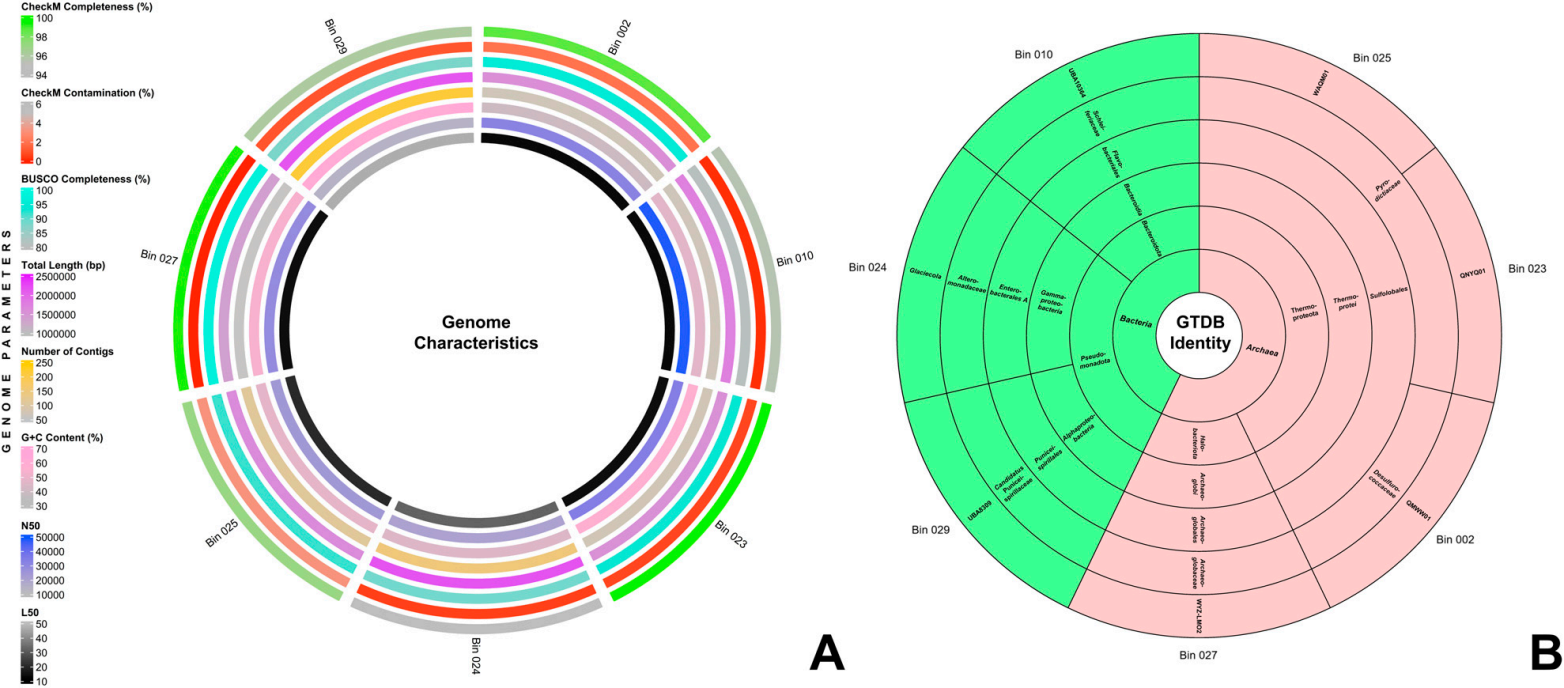
**(A)** Genome characteristics of the seven MAGs. From the innermost to the outermost circle, the rings display the following information: L50, N50, G+C content, contig count, total genome length, BUSCO completeness, CheckM contamination, and CheckM completeness. **(B)** Sunburst chart depicting the GTDB taxonomic identities of the seven MAGs. The innermost ring represents the domain, followed by successive rings for phylum, class, order, family, and genus in the outermost ring.

From the Q30-trimmed metagenomic reads, seven bins were filtered and optimized. Figure 1A provides a summary of their genome characteristics. These seven MAGs exhibited CheckM completeness of over 90% and contamination of under 5%, aligning with MIMAG standards (Bowers et al., 2017). Notably, bins 002, 023, and 027 surpass 98% in CheckM completeness, while bins 010, 023, 024, and 027 reported less than 1% contamination. Additionally, using BUSCO, four of the seven bins scored above 90% completeness. Further quality assessment with QUAST v5.2.0 (Mikheenko et al., 2018) revealed genome sizes ranging from 1.37 to 2.17 Mbp across the bins. Bins 002, 010, 023, and 027 contain fewer than 75 contigs, with bin 027 having the least number at 55 contigs. All bins maintained an L_50_ value below 50, with bins 002 and 010 presenting the lowest at 13. The N_50_ values for all bins were above 14,000, with bin 010 having the highest at 48,078. The GC content of all seven bins ranged from 39.6% to 62.88%. The comprehensive results for CheckM, BUSCO, and QUAST assessments of the seven MAGs are detailed in Supplementary File 1.

The seven bins were taxonomically classified using the Genome Taxonomy Database toolkit (GTDB-tk) v2.3.2 (Chaumeil et al., 2019), and their taxonomic identities are shown in Figure 1B. Four of the seven MAGs were classified under *Archaea*, and three were under *Bacteria*. The MAGs under *Archaea* were divided into two phyla: *Thermoproteota* (Bins 002, 023, 025) and *Halobacteriota* (Bin 027), while the MAGs under *Bacteria* were divided into *Pseudomonadota* (Bins 024, 029) and *Bacteroidota* (Bin 010). Detailed taxonomic identities of all MAGs up to genus level are presented in Supplementary File 2.

**FIGURE 2:**
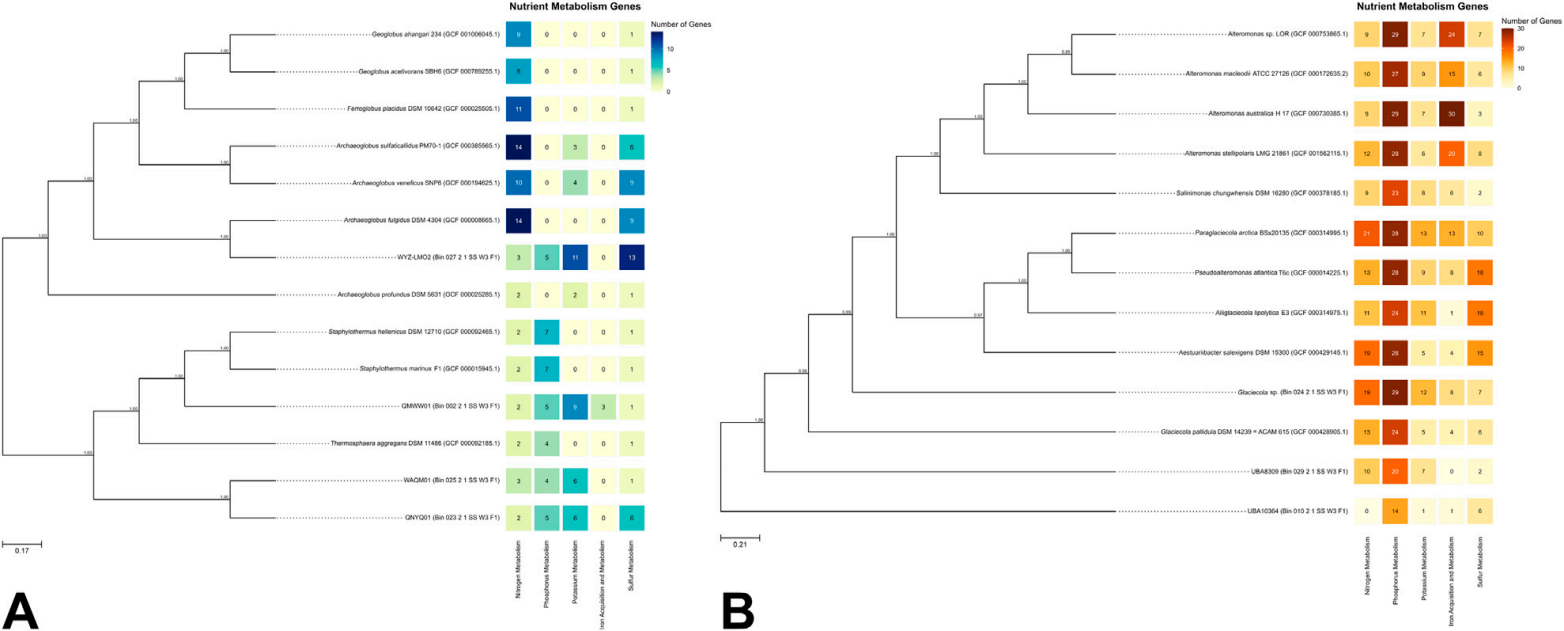
**(A)** Phylogenetic trees of archaeal and **(B)** bacterial MAGs with associated nutrient metabolism gene heatmaps. Node values indicate bootstrap values based on 1,000 replicates. Scale bars represent **(A)** 17% and **(B)** 21% genetic variation per unit of scale length.

Figure 2A presents the top 10 closest genomes to the bins classified under the domain *Archaea*. Figure 2B illustrates the phylogenetic relationships for bins identified under *Bacteria* and their respective top 10 closest genomes.

For archaeal MAGs (Figure 2A), QMWW01 (Bin 002) was found to cluster near the *Staphylothermus* clade, suggesting it may belong to this genus. This is supported by GTDB results (Figure 1B), which identified QMWW01 within the family *Desulfurococcaceae*, the same family as *Staphylothermus*. QNYQ01 (Bin 023) and WAQM01 (Bin 025) formed a monophyletic clade near to *Thermosphaera aggregans*, also within *Desulfurococcaceae* (Anderson et al., 2009). QMWW01, QNYQ01, and WAQM01 clustered with other GenBank genomes, including *Staphylothermus* and *Thermosphaera*, all classified under the order *Sulfolobales*. The *Sulfolobales* genomes displayed genes linked to nitrogen, phosphorus, and sulfur metabolism. *Sulfolobales* is a group of thermoacidophilic *Archaea* where the majority of which are facultatively or obligately chemolithoautotrophic. The ability to metabolize sulfur, whether in its elemental form or reduced inorganic sulfur compounds, enables *Sulfolobales* to grow autotrophically. As a result, it is their most important physiological characteristic (Liu et al., 2021). Additionally, only the *Sulfolobales* MAGs were observed to possess genes associated with potassium metabolism. Notably, QMWW01 uniquely exhibited genes involved in iron acquisition and metabolism within this clade.

On the opposite branch of the tree, WYZ-LMO2 (Bin 027) formed a monophyletic clade with *Archaeoglobus fulgidus*, along with other members of the genera *Ferroglobus*, *Geoglobus*, and other *Archaeoglobus*, all belonging to the family *Archaeoglobaceae* (Brileya and Reysenbach, 2014). All *Archaeoglobaceae* genomes exhibited genes associated with nitrogen and sulfur metabolism. This thermophilic and obligate anaerobic family, found in marine and terrestrial environments, is known for their diverse metabolic capabilities including nitrate and sulfate reduction (Brileya and Reysenbach, 2014). Notably, WYZ-LMO2 was the only genome within the clade to possess genes associated with phosphorus metabolism. Lastly, no genome within the clade exhibited genes for iron acquisition and metabolism.

For bacterial MAGs (Figure 2B), *Glacieola* sp. (Bin 024) and UBA8309 (Bin 029) were identified as closely related to the GenBank genome *Glaciecola pallidula* DSM 14239. All three genomes were classified under the phylum *Pseudomonadota* (syn. *Proteobacteria*). Both *Glaciecola pallidula* DSM 14239 and *Glaciecola* sp. contained genes associated with all the types of metabolism genes analyzed in this report. Members of the genus *Glaciecola* are known to inhabit various marine environments (Bian et al., 2011; Qin et al., 2014), including seawater similar to the sample source in this study. This genus was also recognized for its ability to break down biopolymers such as cellulose, chitin, and xylan (Qin et al., 2014), suggesting a significant role in organic matter degradation and carbon cycling in marine environments. For UBA8309, the MAG was classified under the family *Candidatus* Puniceispirillaceae. *Puniceispirillum marinum* IMCC1322, which belongs to the same family, is known for its metabolic generalism in oceanic nutrient cycling and possesses genes associated with dimethylsulfoniopropionate (DMSP), which may play a role in marine sulfur cycling (Oh et al., 2010). This suggests that UBA8309 may also play a role in nutrient cycling in SGD areas.

UBA10364 (Bin 010) was identified as the only MAG classified under *Bacteroidota* and was the most distantly related bin compared to the GenBank bacterial genomes. The MAG falls under the order *Flavobacteriales*. Members of this group are known to metabolize sulfur-containing substances and participate in the sulfur cycle (Wang et al., 2023). They also produce extracellular hydrolases to degrade macromolecular organic substances (Wang et al., 2023). The presence of sulfur metabolism genes in UBA10364 suggests a role in sulfur cycling, and the detection of genes related to phosphorus, potassium, and iron metabolism indicates potential involvement in cycling these elements as well.

The nutrient metabolism genes identified in the bins were further analyzed (Supplementary File 3). For nitrogen metabolism, the bins were found to contain genes associated with nitrate and nitrite ammonification, as well as ammonia assimilation. In the case of phosphorus metabolism, genes involved in phosphate metabolism, high-affinity phosphate transport, phosphate regulon, and polyphosphate synthesis were detected. For potassium metabolism, the identified genes included those related to the glutathione-regulated potassium-efflux system, hyperosmotic potassium uptake, and potassium homeostasis. For sulfur metabolism, genes linked to galactosylceramide and sulfatide metabolism, sulfite reduction, and thioredoxin-disulfide reductase were observed. Lastly, within the iron acquisition and metabolism category, genes encoding encapsulating proteins for peroxidase enzymes, iron acquisition mechanisms, and the hemin transport system were identified. In addition to the RAST results, the DRAM analysis (Supplementary File 4) of the bins revealed a diverse array of nutrient metabolism genes. These included genes involved in carbon, nitrogen, and sulfur metabolism, as well as genes associated with arsenate and mercury reduction, methanogenesis, and alcohol production.

The diverse metabolic capabilities of the recovered bacterial and archaeal MAGs, supported by annotated genes and their taxonomic identities, suggest that these putative microorganisms may play a crucial role in biogeochemical cycling in SGD areas of Mabini, Batangas. This conclusion is further supported by various studies highlighting the metabolic functions of similar taxa in nutrient cycling.

Functional COGs were identified across all MAGs using eggNOG. The analysis revealed that the category with the highest relative frequency of COGs was “Unknown function” (S) (Supplementary File 5; Supplementary Figure S1). This observation aligns with individual MAG results, where the most frequently assigned COG in all bins also belonged to the “Unknown function” category (Supplementary File 5; Supplementary Figures S2–S8). This finding suggests that many COGs within the bins require additional data for proper classification. In addition to the “Unknown function” category, several functional COGs were found to have relative frequencies exceeding 5%, including J (translation, ribosomal structure, and biogenesis), C (energy production and conversion), E (amino acid transport and metabolism), H (coenzyme transport and metabolism), and L (replication, recombination, and repair). The abundance of these functional genes suggests that the microorganisms in the vent water heavily rely on these processes for survival, particularly in the SGD environment.

In addition to the analysis of the nutrient metabolism genes and COGs, the presence of BGCs within the MAGs was assessed, as detailed in Supplementary File 6. Using antiSMASH, it was found that only QNYQ01 (Bin 023) and WYZ-LMO2 (Bin 027) lacked detectable BGCs. In contrast, the remaining MAGs exhibited BGCs associated with ectoine, ribosomally synthesized, and post-translationally modified peptides (RiPP)-like compounds, terpene, and redox-cofactors. Among these, *Glacieola* sp. (Bin 024) contained the highest number of BGCs, including those related to ectoine, RiPP-like compounds, and terpene. Notably, RiPP-like and terpene-related BGCs were the most prevalent across all MAGs. RiPP-like compounds, previously identified as bacteriocin-encoding genes (Blin et al., 2021), have been studied for their anticancer and antibiotic applications (Negash and Tsehai, 2020; Thapar and Kumar Salooja, 2023). Similarly, terpenes are renowned for their diverse biological activities, including antiplasmodial, antiviral, anticancer, and antidiabetic properties (Cox-Georgian et al., 2019).

The identification of these BGCs in putative microbes from vent water at SGD sites in Mabini, Batangas, suggests their potential biotechnological and pharmaceutical applications. Although these microorganisms remain unculturable, future advances in cultivation and isolation techniques may enable us to unlock their potential, leading to the discovery of novel therapeutic compounds and other valuable bioactive agents.

## Data Availability

The datasets presented in this study can be found in online repositories. The names of the repository/repositories and accession number(s) can be found below: https://www.ncbi.nlm.nih.gov/bioproject/1111281 https://zenodo.org/records/14490404 https://narrative.kbase.us/narrative/202183.

## References

[B1] Adyasari D. Hassenrück C. Oehler T. Sabdaningsih A. Moosdorf N. (2019). Microbial community structure associated with submarine groundwater discharge in Northern Java (Indonesia). Sci. Total Environ. 689, 590–601. 10.1016/j.scitotenv.2019.06.193 31279205

[B2] Alneberg J. Bjarnason B. S. de Bruijn I. Schirmer M. Quick J. Ijaz U. Z. (2014). Binning metagenomic contigs by coverage and composition. Nat. Methods 11 (11), 1144–1146. 10.1038/nmeth.3103 25218180

[B3] Anderson I. J. Sun H. Lapidus A. Copeland A. Glavina Del Rio T. Tice H. (2009). Complete genome sequence of *Staphylothermus marinus* Stetter and Fiala 1986 type strain F1. Stand. Genomic Sci. 1 (2), 183–188. 10.4056/sigs.30527 21304655 PMC3035234

[B4] Arkin A. P. Cottingham R. W. Henry C. S. Harris N. L. Stevens R. L. Maslov S. (2018). KBase: the United States department of energy systems biology knowledgebase. Nat. Biotechnol. 36 (7), 566–569. 10.1038/nbt.4163 29979655 PMC6870991

[B5] Aziz R. K. Bartels D. Best A. A. DeJongh M. Disz T. Edwards R. A. (2008). The RAST server: rapid annotations using subsystems technology. BMC Genomics 9 (1), 75. 10.1186/1471-2164-9-75 18261238 PMC2265698

[B6] Bernard R. Mortazavi B. Wang L. Ortmann A. MacIntyre H. Burnett W. (2014). Benthic nutrient fluxes and limited denitrification in a sub-tropical groundwater-influenced Coastal Lagoon. Mar. Ecol. Prog. Ser. 504, 13–26. 10.3354/meps10783

[B7] Bian F. Qin Q.-L. Xie B.-B. Shu Y.-L. Zhang X.-Y. Yu Y. (2011). Complete genome sequence of seawater bacterium *Glaciecola nitratireducens* FR1064^T^ . J. Bacteriol. 193 (24), 7006–7007. 10.1128/jb.06296-11 22123761 PMC3232840

[B8] Blin K. Shaw S. Augustijn H. E. Reitz Z. L. Biermann F. Alanjary M. (2023). antiSMASH 7.0: new and improved predictions for detection, regulation, chemical structures and visualisation. Nucleic Acids Res. 51 (W1), W46–W50. 10.1093/nar/gkad344 37140036 PMC10320115

[B9] Blin K. Shaw S. Kloosterman A. M. Charlop-Powers Z. van Wezel G. P. Medema M. H. (2021). antiSMASH 6.0: improving cluster detection and comparison capabilities. Nucleic Acids Res. 49 (W1), W29–W35. 10.1093/nar/gkab335 33978755 PMC8262755

[B10] Bolger A. M. Lohse M. Usadel B. (2014). Trimmomatic: a flexible trimmer for Illumina sequence data. Bioinformatics 30 (15), 2114–2120. 10.1093/bioinformatics/btu170 24695404 PMC4103590

[B11] Bowers R. M. Kyrpides N. C. Stepanauskas R. Harmon-Smith M. Doud D. Reddy T. B. (2017). Minimum information about a single amplified genome (MISAG) and a metagenome-assembled genome (MIMAG) of bacteria and archaea. Nat. Biotechnol. 35 (8), 725–731. 10.1038/nbt.3893 28787424 PMC6436528

[B12] Brileya K. Reysenbach A.-L. (2014). The class Archaeoglobi. Prokaryotes, 15–23. 10.1007/978-3-642-38954-2_323

[B13] Burnett W. C. Aggarwal P. K. Aureli A. Bokuniewicz H. Cable J. E. Charette M. A. (2006). Quantifying submarine groundwater discharge in the coastal zone via multiple methods. Sci. Total Environ. 367 (2–3), 498–543. 10.1016/j.scitotenv.2006.05.009 16806406

[B14] Burnett W. C. Cable J. E. Corbett D. R. (2003). Radon tracing of submarine groundwater discharge in coastal environments. Land Mar. Hydrogeology, 25–43. 10.1016/b978-044451479-0/50015-7

[B15] Cantalapiedra C. P. Hernández-Plaza A. Letunic I. Bork P. Huerta-Cepas J. (2021). EggNOG-mapper V2: functional annotation, Orthology assignments, and domain prediction at the metagenomic scale. Mol. Biol. Evol. 38 (12), 5825–5829. 10.1093/molbev/msab293 34597405 PMC8662613

[B16] Cardenas M. B. Rodolfo R. S. Lapus M. R. Cabria H. B. Fullon J. Gojunco G. R. (2020). Submarine groundwater and vent discharge in a volcanic area associated with coastal acidification. Geophys. Res. Lett. 47 (1). 10.1029/2019gl085730

[B17] Chaumeil P.-A. Mussig A. J. Hugenholtz P. Parks D. H. (2019). GTDB-TK: a toolkit to classify genomes with the Genome Taxonomy Database. Bioinformatics 36 (6), 1925–1927. 10.1093/bioinformatics/btz848 31730192 PMC7703759

[B18] Chen X. Cai R. Zhuo X. Chen Q. He C. Sun J. (2023). Niche differentiation of microbial community shapes vertical distribution of recalcitrant dissolved organic matter in deep-sea sediments. Environ. Int. 178, 108080. 10.1016/j.envint.2023.108080 37429058

[B19] Cho H.-M. Kim G. Kwon E. Y. Moosdorf N. Garcia-Orellana J. Santos I. R. (2018). Radium tracing nutrient inputs through submarine groundwater discharge in the Global Ocean. Sci. Rep. 8 (1), 2439. 10.1038/s41598-018-20806-2 29403050 PMC5799265

[B20] Cox-Georgian D. Ramadoss N. Dona C. Basu C. (2019). Therapeutic and medicinal uses of Terpenes. Med. Plants, 333–359. 10.1007/978-3-030-31269-5_15

[B21] Griebler C. Lueders T. (2009). Microbial Biodiversity in groundwater ecosystems. Freshw. Biol. 54 (4), 649–677. 10.1111/j.1365-2427.2008.02013.x

[B22] Gu Z. Gu L. Eils R. Schlesner M. Brors B. (2014). Circlize implements and enhances circular visualization in R. Bioinformatics 30 (19), 2811–2812. 10.1093/bioinformatics/btu393 24930139

[B23] He Y. Li M. Perumal V. Feng X. Fang J. Xie J. (2016). Genomic and enzymatic evidence for acetogenesis among multiple lineages of the archaeal phylum Bathyarchaeota widespread in marine sediments. Nat. Microbiol. 1 (4), 16035. 10.1038/nmicrobiol.2016.35 27572832

[B24] Huerta-Cepas J. Szklarczyk D. Heller D. Hernández-Plaza A. Forslund S. K. Cook H. (2018). Eggnog 5.0: a hierarchical, functionally and phylogenetically annotated orthology resource based on 5090 organisms and 2502 viruses. Nucleic Acids Res. 47 (D1), D309–D314. 10.1093/nar/gky1085 PMC6324079 30418610

[B25] Kang D. Li F. Kirton E. Thomas A. Egan R. An H. (2019). MetaBAT 2: an adaptive binning algorithm for robust and efficient genome reconstruction from Metagenome Assemblies. PeerJ 7, e7359. 10.7717/peerj.7359 31388474 PMC6662567

[B26] Knee K. L. Paytan A. (2011). 4.08 - submarine groundwater discharge: a source of nutrients, metals, and pollutants to the coastal ocean. Treatise Estuar. Coast. Sci., 205–233. 10.1016/b978-0-12-374711-2.00410-1

[B27] Li D. Luo R. Liu C.-M. Leung C.-M. Ting H.-F. Sadakane K. (2016). Megahit v1.0: a fast and scalable metagenome assembler driven by advanced methodologies and community practices. Methods 102, 3–11. 10.1016/j.ymeth.2016.02.020 27012178

[B28] Liu L.-J. Jiang Z. Wang P. Qin Y.-L. Xu W. Wang Y. (2021). Physiology, taxonomy, and sulfur metabolism of the Sulfolobales, an order of thermoacidophilic archaea. Front. Microbiol. 12, 768283. 10.3389/fmicb.2021.768283 34721370 PMC8551704

[B29] Mangoma N. Zhou N. Ncube T. (2024). Metagenome-assembled genomes provide insight into the microbial taxonomy and ecology of the Buhera soda pans, Zimbabwe. PLOS ONE 19 (12), e0299620. 10.1371/journal.pone.0299620 39621710 PMC11611182

[B30] Mikheenko A. Prjibelski A. Saveliev V. Antipov D. Gurevich A. (2018). Versatile genome assembly evaluation with QUAST-LG. Bioinformatics 34 (13), i142–i150. 10.1093/bioinformatics/bty266 29949969 PMC6022658

[B31] Moore W. S. (2010). The effect of submarine groundwater discharge on the Ocean. Annu. Rev. Mar. Sci. 2 (1), 59–88. 10.1146/annurev-marine-120308-081019 21141658

[B32] Negash A. W. Tsehai B. A. (2020). Current applications of bacteriocin. Int. J. Microbiol. 2020, 4374891–4374897. 10.1155/2020/4374891 33488719 PMC7803181

[B33] Nurk S. Meleshko D. Korobeynikov A. Pevzner P. A. (2017). metaSPAdes: a new versatile metagenomic assembler. Genome Res. 27 (5), 824–834. 10.1101/gr.213959.116 28298430 PMC5411777

[B34] Oh H.-M. Kwon K. K. Kang I. Kang S. G. Lee J.-H. Kim S.-J. (2010). Complete genome sequence of “*Candidatus Puniceispirillum marinum*” IMCC1322, a representative of the SAR116 clade in the *Alphaproteobacteria* . J. Bacteriol. 192 (12), 3240–3241. 10.1128/jb.00347-10 20382761 PMC2901696

[B35] Overbeek R. Olson R. Pusch G. D. Olsen G. J. Davis J. J. Disz T. (2013). The SEED and the rapid annotation of microbial genomes using subsystems technology (RAST). Nucleic Acids Res. 42 (D1), D206–D214. 10.1093/nar/gkt1226 24293654 PMC3965101

[B36] Overholt W. A. Hölzer M. Geesink P. Diezel C. Marz M. Küsel K. (2020). Inclusion of oxford nanopore long reads improves all microbial and viral metagenome‐assembled genomes from a complex aquifer system. Environ. Microbiol. 22 (9), 4000–4013. 10.1111/1462-2920.15186 32761733

[B37] Parks D. H. Imelfort M. Skennerton C. T. Hugenholtz P. Tyson G. W. (2015). Checkm: assessing the quality of microbial genomes recovered from isolates, single cells, and metagenomes. Genome Res. 25 (7), 1043–1055. 10.1101/gr.186072.114 25977477 PMC4484387

[B38] Peng Y. Leung H. C. Yiu S. M. Chin F. Y. (2012). IDBA-UD: a *de novo* assembler for single-cell and metagenomic sequencing data with highly uneven depth. Bioinformatics 28 (11), 1420–1428. 10.1093/bioinformatics/bts174 22495754

[B39] Prjibelski A. Antipov D. Meleshko D. Lapidus A. Korobeynikov A. (2020). Using SPAdes *de novo* assembler. Curr. Protoc. Bioinforma. 70 (1), e102. 10.1002/cpbi.102 32559359

[B40] Purkamo L. von Ahn C. M. Jilbert T. Muniruzzaman M. Bange H. W. Jenner A.-K. (2022). Impact of submarine groundwater discharge on biogeochemistry and microbial communities in Pockmarks. Geochimica Cosmochimica Acta 334, 14–44. 10.1016/j.gca.2022.06.040

[B41] Qin Q.-L. Xie B.-B. Yu Y. Shu Y.-L. Rong J.-C. Zhang Y.-J. (2014). Comparative genomics of the marine bacterial genus *Glaciecola* reveals the high degree of genomic diversity and genomic characteristic for cold adaptation. Environ. Microbiol. 16 (6), 1642–1653. 10.1111/1462-2920.12318 25009843

[B42] RStudio Team (2024). *RStudio: Integrated development environment for R*. RStudio. PBC. Available at: https://www.rstudio.com.

[B43] Ruiz-González C. Rodellas V. Garcia-Orellana J. (2021). The microbial dimension of submarine groundwater discharge: current challenges and Future Directions. FEMS Microbiol. Rev. 45 (5), fuab010. 10.1093/femsre/fuab010 33538813 PMC8498565

[B44] Santos I. R. Chen X. Lecher A. L. Sawyer A. H. Moosdorf N. Rodellas V. (2021). Submarine groundwater discharge impacts on coastal nutrient biogeochemistry. Nat. Rev. Earth and Environ. 2 (5), 307–323. 10.1038/s43017-021-00152-0

[B45] Schlüter M. Sauter E. J. Andersen C. E. Dahlgaard H. Dando P. R. (2004). Spatial distribution and budget for submarine groundwater discharge in eckernförde bay (western baltic sea). Limnol. Oceanogr. 49 (1), 157–167. 10.4319/lo.2004.49.1.0157

[B46] Shaffer M. Borton M. A. McGivern B. B. Zayed A. A. La Rosa S. L. Solden L. M. (2020). Dram for distilling microbial metabolism to automate the curation of microbiome function. Nucleic Acids Res. 48 (16), 8883–8900. 10.1093/nar/gkaa621 32766782 PMC7498326

[B47] Sieber C. M. Probst A. J. Sharrar A. Thomas B. C. Hess M. Tringe S. G. (2018). Recovery of genomes from metagenomes via a dereplication, aggregation and scoring strategy. Nat. Microbiol. 3 (7), 836–843. 10.1038/s41564-018-0171-1 29807988 PMC6786971

[B48] Simão F. A. Waterhouse R. M. Ioannidis P. Kriventseva E. V. Zdobnov E. M. (2015). BUSCO: assessing genome assembly and annotation completeness with single-copy orthologs. Bioinformatics 31 (19), 3210–3212. 10.1093/bioinformatics/btv351 26059717

[B49] Smith M. B. Rocha A. M. Smillie C. S. Olesen S. W. Paradis C. Wu L. (2015). Natural bacterial communities serve as quantitative geochemical biosensors. mBio 6 (3), e00326. 10.1128/mbio.00326-15 25968645 PMC4436078

[B50] Thapar P. Kumar Salooja M. (2023). Bacteriocins: applications in food preservation and therapeutics. Lact. - A Multifunct. Genus. 10.5772/intechopen.106871

[B51] Wang Y. Wang L. Liu Y. Su S. Hao W. (2023). Marine bacterial communities in the xisha islands, South China sea. Diversity 15 (7), 865. 10.3390/d15070865

[B52] Wickham H. (2016). ggplot2: elegant graphics for data analysis. New York: Springer-Verlag. Available at: https://ggplot2.tidyverse.org (ISBN 978-3-319-24277-4.

[B53] Wickham H. (2023). stringr: simple, consistent wrappers for common string operations (R package version 1.5.1). Available at: https://stringr.tidyverse.org.

[B54] Wickham H. François R. Henry L. Müller K. Vaughan D. (2023). dplyr: a grammar of data manipulation (R package version 1.1.4). Available at: https://github.com/tidyverse/dplyr.

[B55] Wickham H. Hester J. Bryan J. (2024). readr: read rectangular text data (R package) version 2.1.5. Available at: https://readr.tidyverse.org.

[B56] Wu Y.-W. Simmons B. A. Singer S. W. (2015). MaxBin 2.0: an automated binning algorithm to recover genomes from multiple metagenomic datasets. Bioinformatics 32 (4), 605–607. 10.1093/bioinformatics/btv638 26515820

[B57] Yu G. Smith D. K. Zhu H. Guan Y. Lam T. T. (2016). *ggtree*: an R package for visualization and annotation of phylogenetic trees with their covariates and other associated data. Methods Ecol. Evol. 8 (1), 28–36. 10.1111/2041-210x.12628

