# Supplementary material for: Exploring the taxonomical and functional profiles of marine microorganisms in Submarine Groundwater Discharge vent water from Mabini, Batangas, Philippines through metagenome-assembled genomes
Source: Front Genet. 2025 Feb 10;16:1522253. doi: 10.3389/fgene.2025.1522253 (PMC11868764; doi:10.3389/fgene.2025.1522253)
Supplement: Supplementary file 2 [file Table1.docx]

**Supplementary File 1**

**Table 1.** Assembly characteristics of the seven generated MAGs. A deeper shade of green indicates higher quality values relative to other bins.

| **Bins** | **GTDB Identity** | **CheckM (%)** | | **BUSCO (%)** | **QUAST** | | | | |
| --- | --- | --- | --- | --- | --- | --- | --- | --- | --- |
|  |  | **Completeness** | **Contamination** | **Completeness** | **Total Length (bp)** | **Number of Contigs** | **GC  Content (%)** | **N50** | **L50** |
| Bin 002 | QMWW01 | 98.73 | 1.93 | 95.10 | 1,575,296 | 70 | 39.60 | 32,023 | 13 |
| Bin 010 | UBA10364 | 95.43 | 0.18 | 80.80 | 1,732,353 | 69 | 46.63 | 48,078 | 13 |
| Bin 023 | QNYQ01 | 99.68 | 0.63 | 93.30 | 1,555,736 | 73 | 57.01 | 33,854 | 14 |
| Bin 024 | *Glaciecola* sp. | 92.58 | 0.51 | 88.70 | 2,171,158 | 150 | 44.87 | 19,953 | 32 |
| Bin 025 | WAQM01 | 96.99 | 3.16 | 91.80 | 1,617,595 | 110 | 47.34 | 23,710 | 20 |
| Bin 027 | WYZ-LMO2 | 99.35 | 0.00 | 97.40 | 1,377,565 | 55 | 53.77 | 29,010 | 14 |
| Bin 029 | UBA8309 | 96.08 | 1.02 | 87.90 | 2,131,358 | 214 | 62.88 | 14,275 | 47 |
