# Supplementary material for: Exploring the taxonomical and functional profiles of marine microorganisms in Submarine Groundwater Discharge vent water from Mabini, Batangas, Philippines through metagenome-assembled genomes
Source: Front Genet. 2025 Feb 10;16:1522253. doi: 10.3389/fgene.2025.1522253 (PMC11868764; doi:10.3389/fgene.2025.1522253)
Supplement: Supplementary file 3 [file Table5.docx]

**Supplementary File 5**

**Clusters of Orthologous Genes Detected in the 7 MAGs**


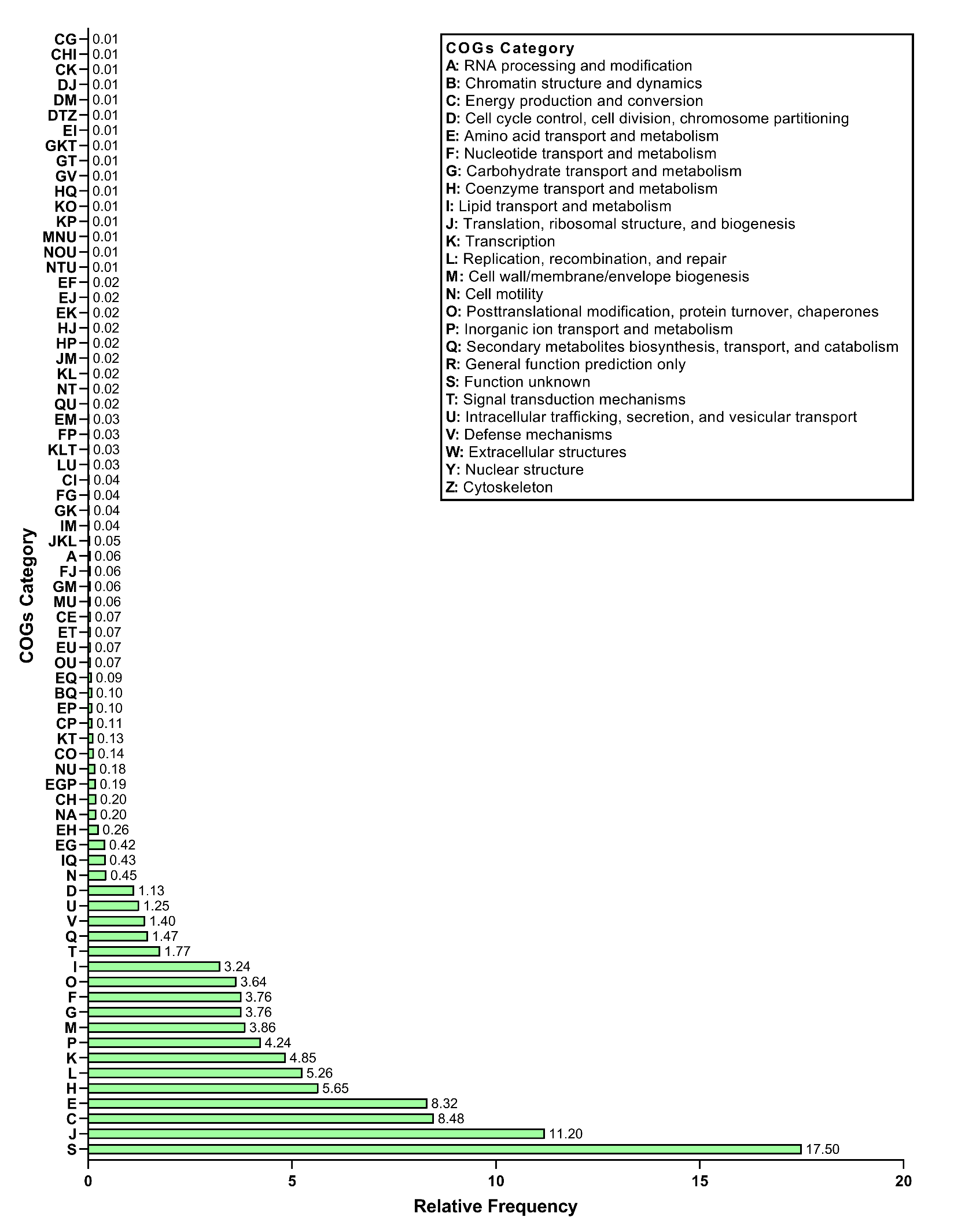


**Figure S1.** Detected COGs in all of the 2_1_SS_W3_F1 Bins.


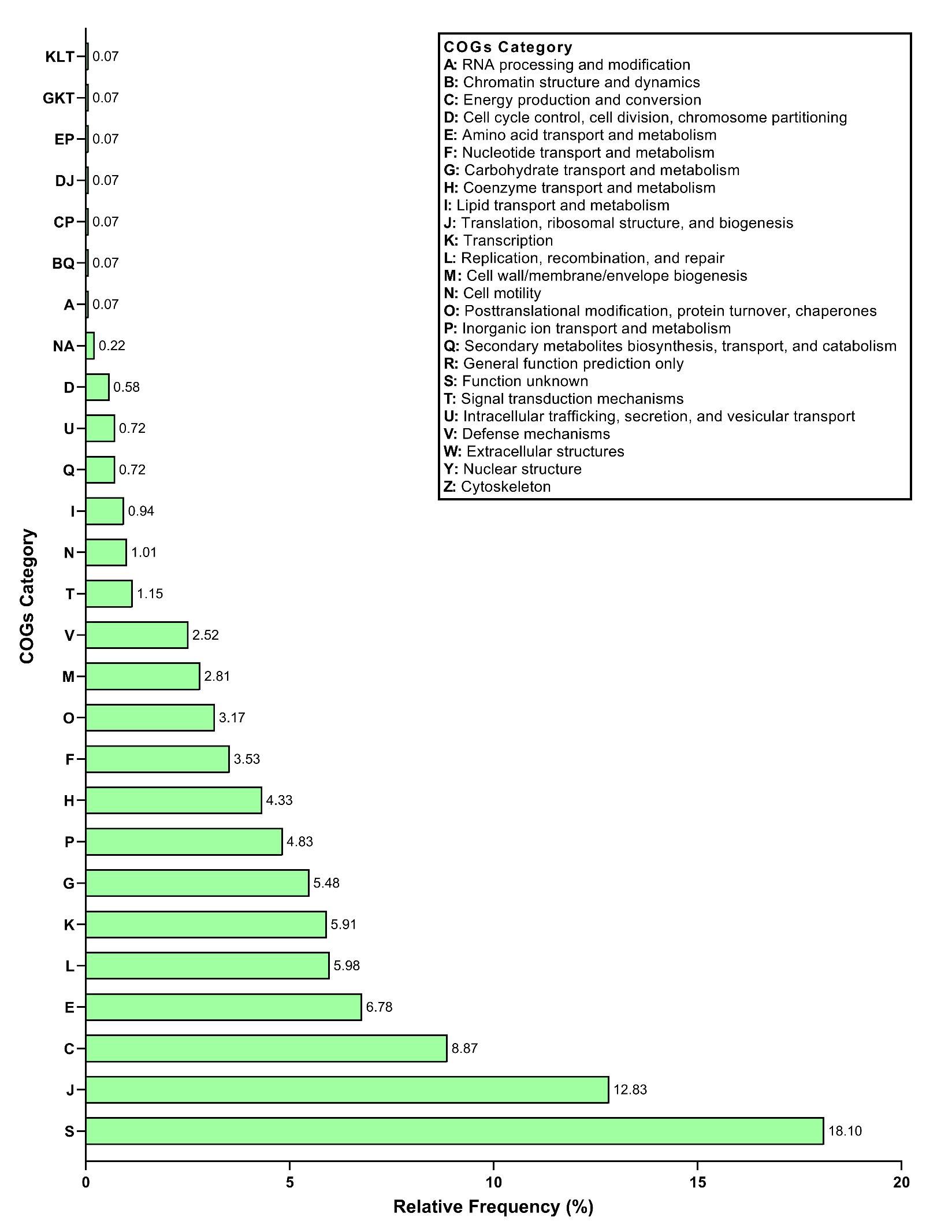


**Figure S2.** Detected COGs in Bin 002 (QMWW01).


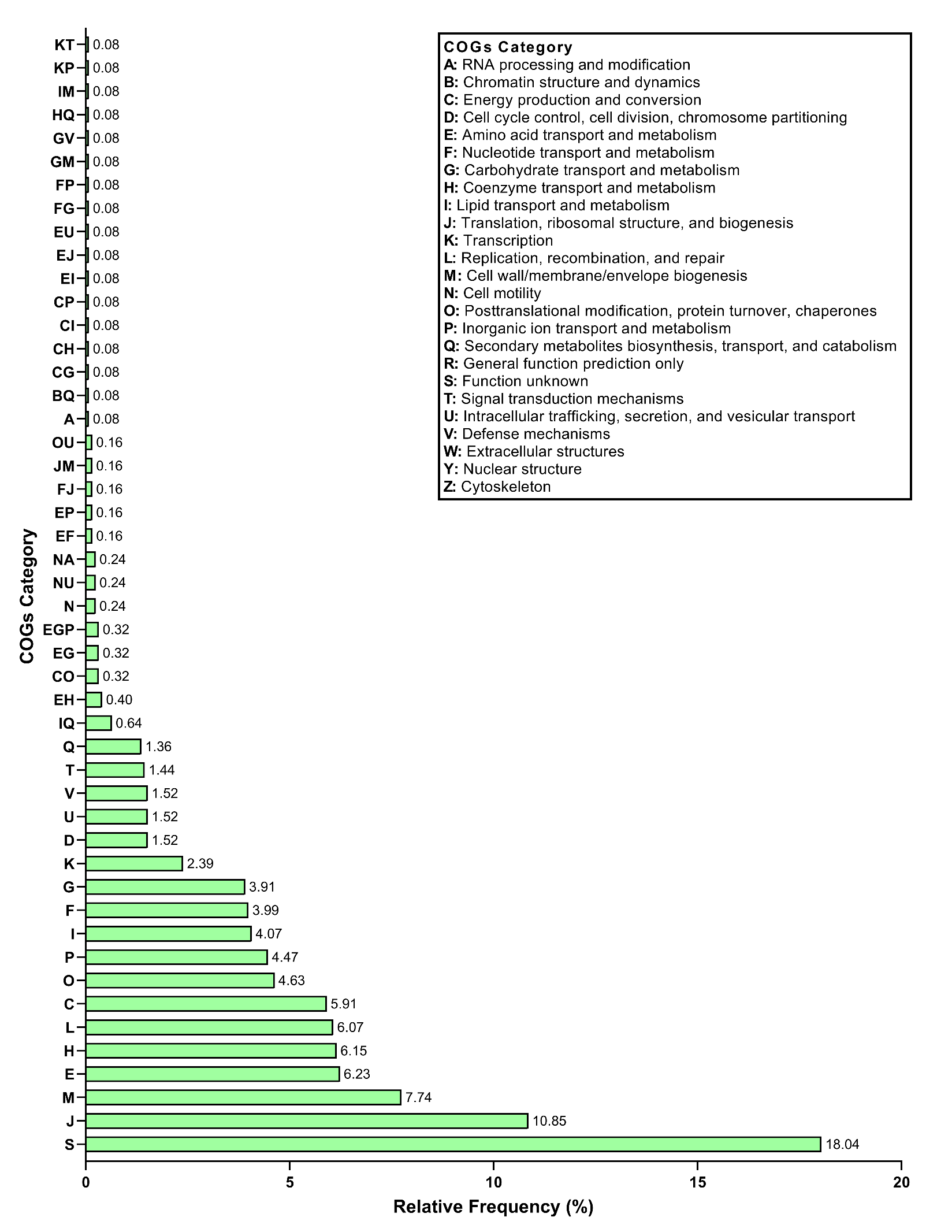


**Figure S3.** Detected COGs in Bin 010 (UBA10364).

**
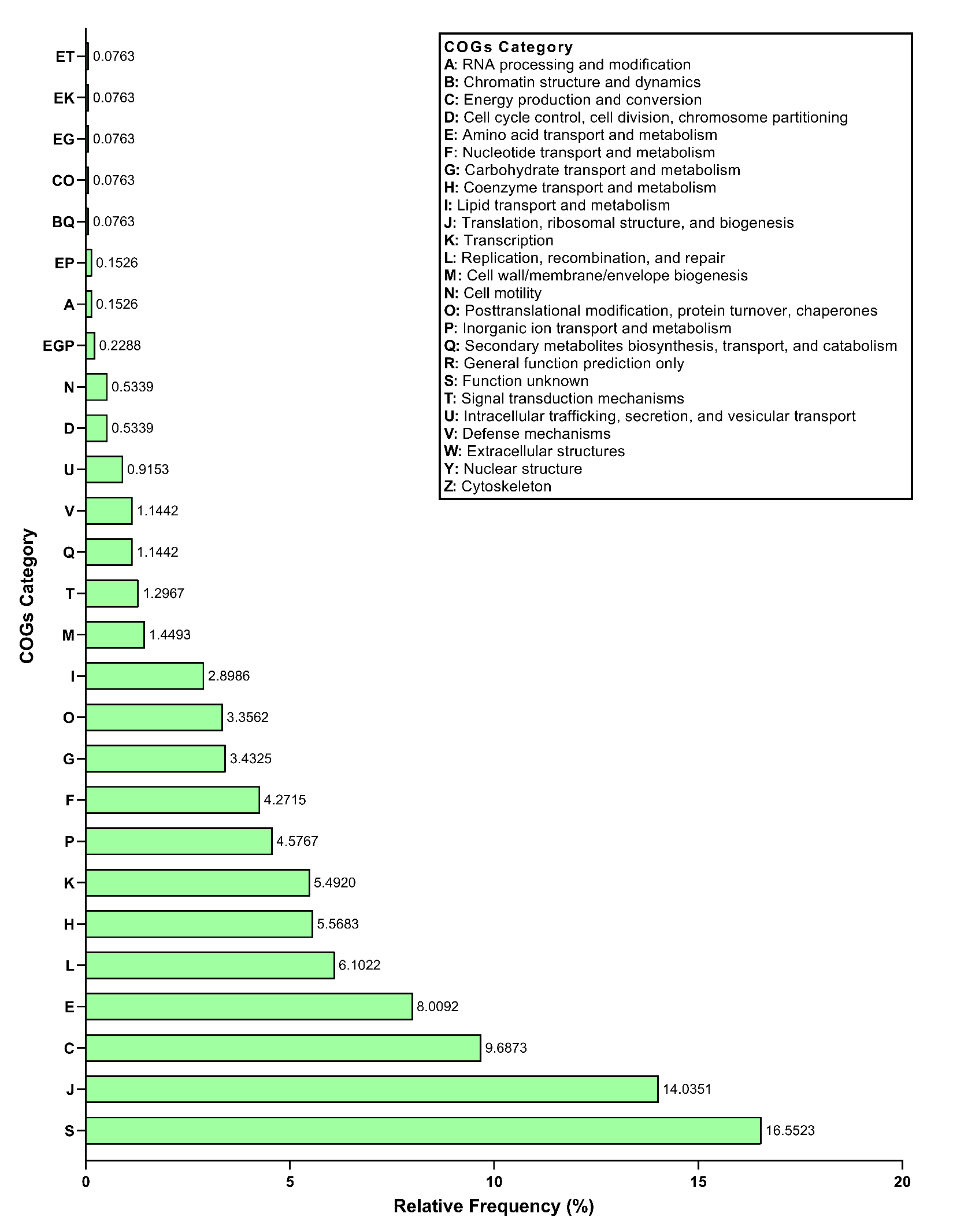
**

**Figure S4.** Detected COGs in Bin 023 (QNYQ01).

**
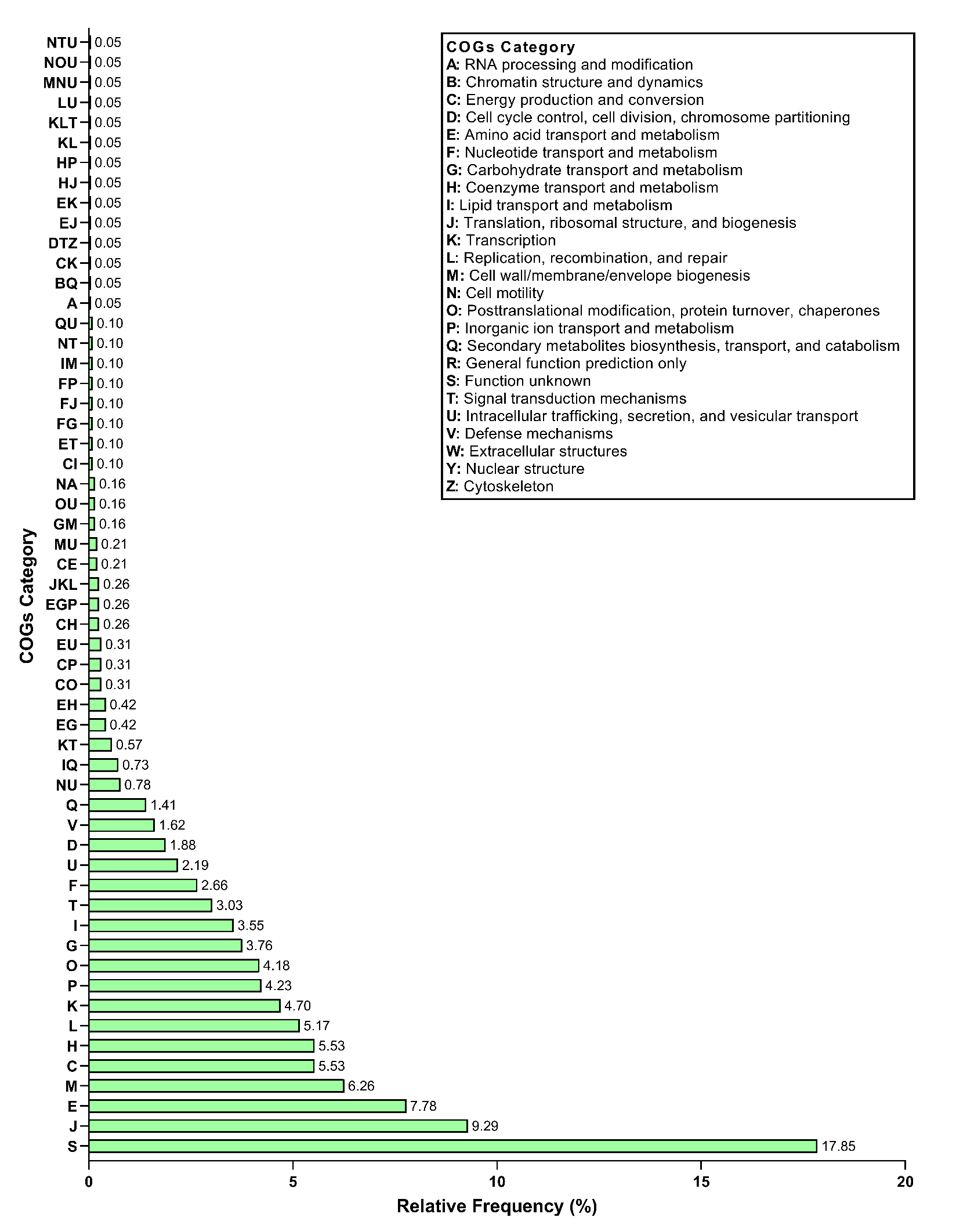
**

**Figure S5.** Detected COGs in Bin 024 (*Glacieola* sp.).


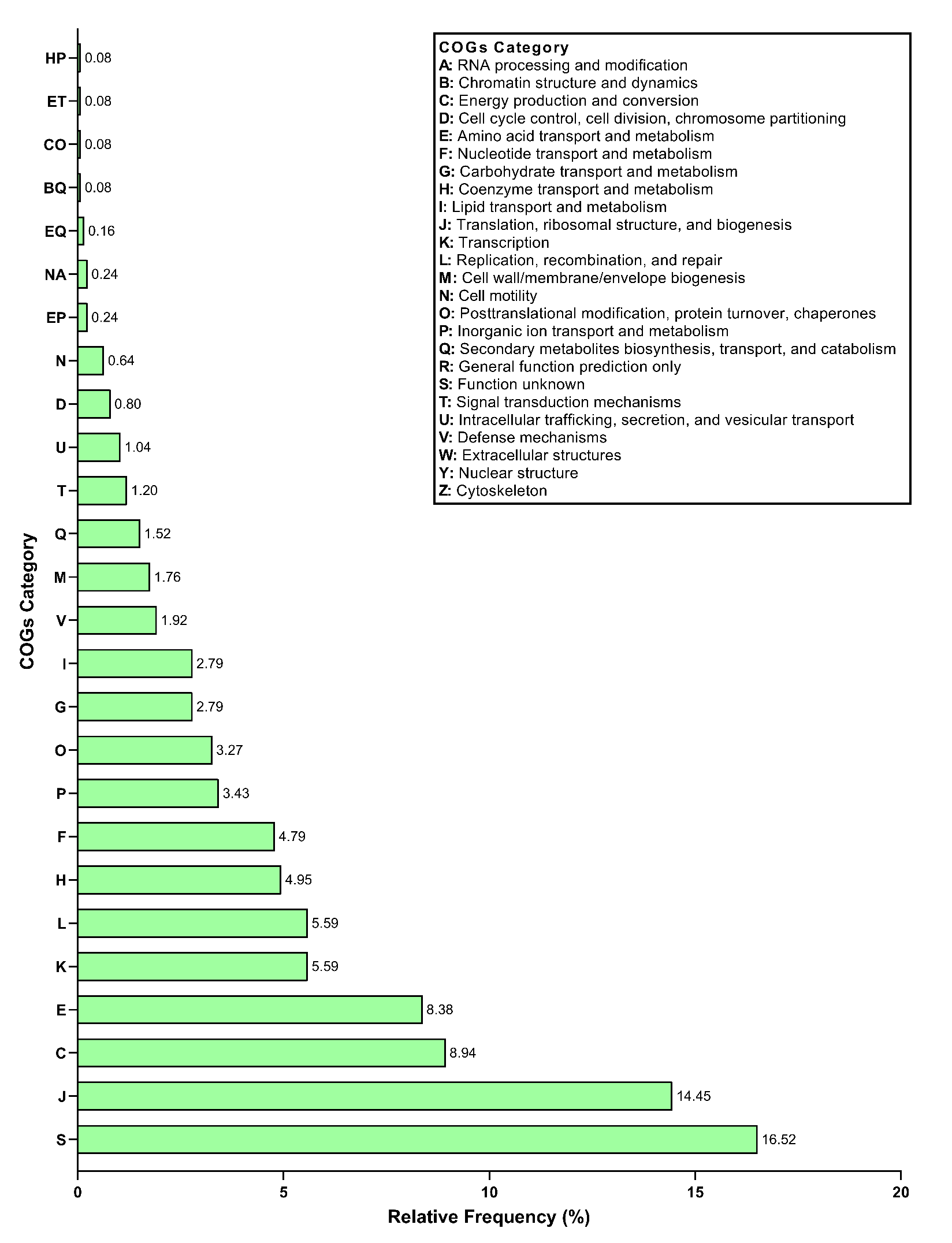


**Figure S6.** Detected COGs in Bin 025 (WAQM01).


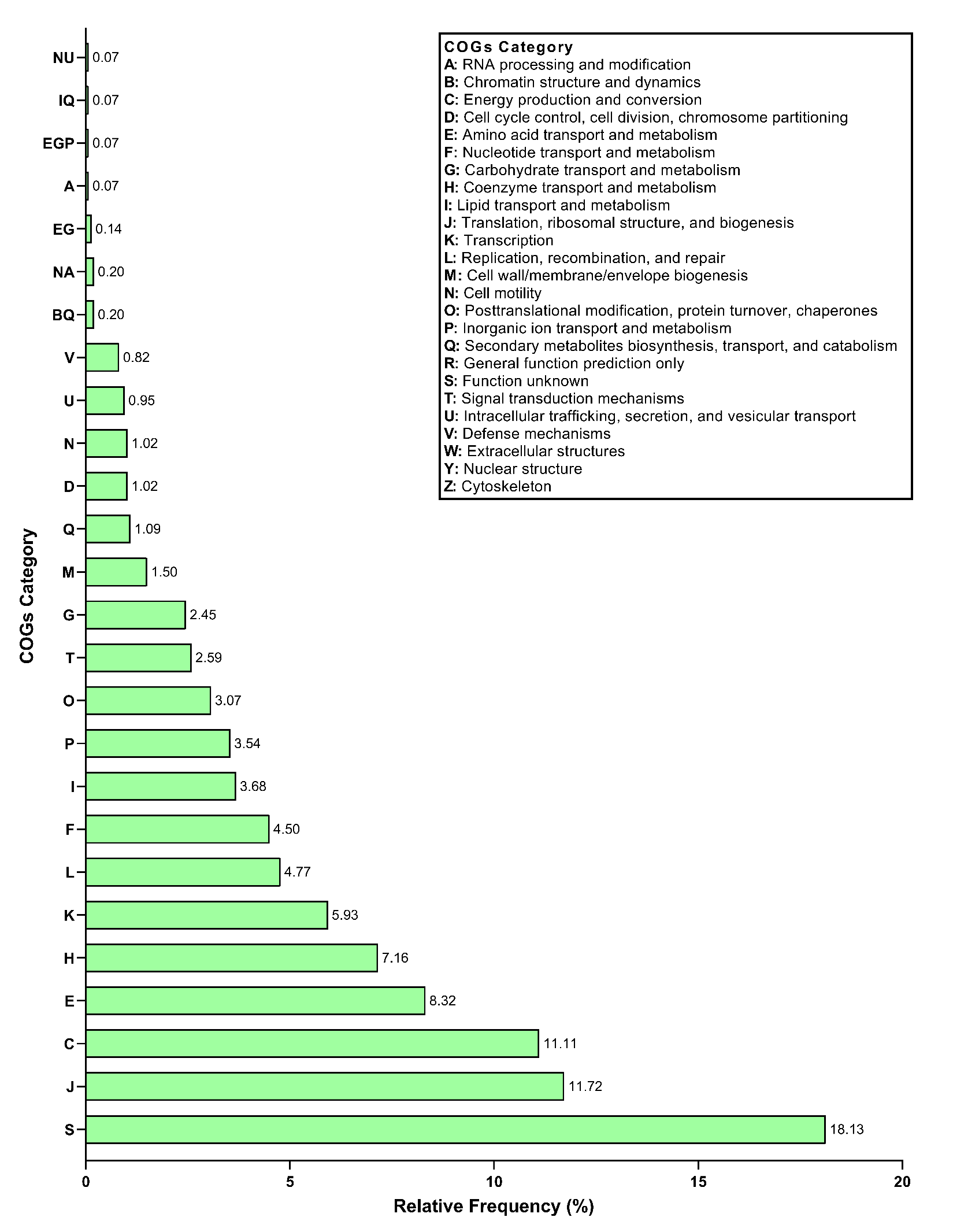


**Figure S7.** Detected COGs in Bin 027 (WYZ-LMO2).


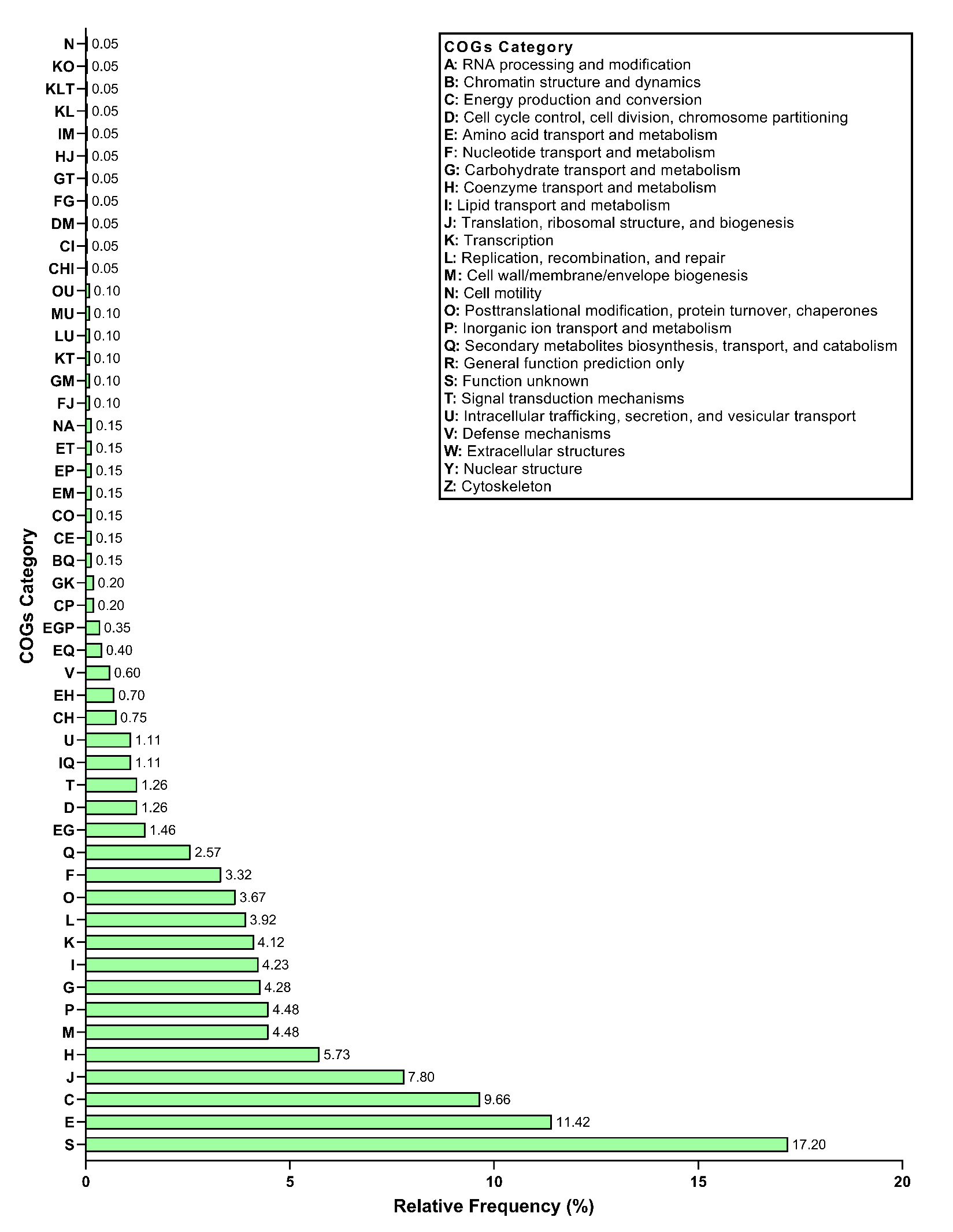


**Figure S8.** Detected COGs in Bin 029 (UBA8309).
