# Supplementary material for: Exploring the taxonomical and functional profiles of marine microorganisms in Submarine Groundwater Discharge vent water from Mabini, Batangas, Philippines through metagenome-assembled genomes
Source: Front Genet. 2025 Feb 10;16:1522253. doi: 10.3389/fgene.2025.1522253 (PMC11868764; doi:10.3389/fgene.2025.1522253)
Supplement: Supplementary file 4 [file Table3.docx]

**Supplementary File 3**

**Detected Nutrient Metabolism Genes in the 2_1_SS_W3_F1 Bins**


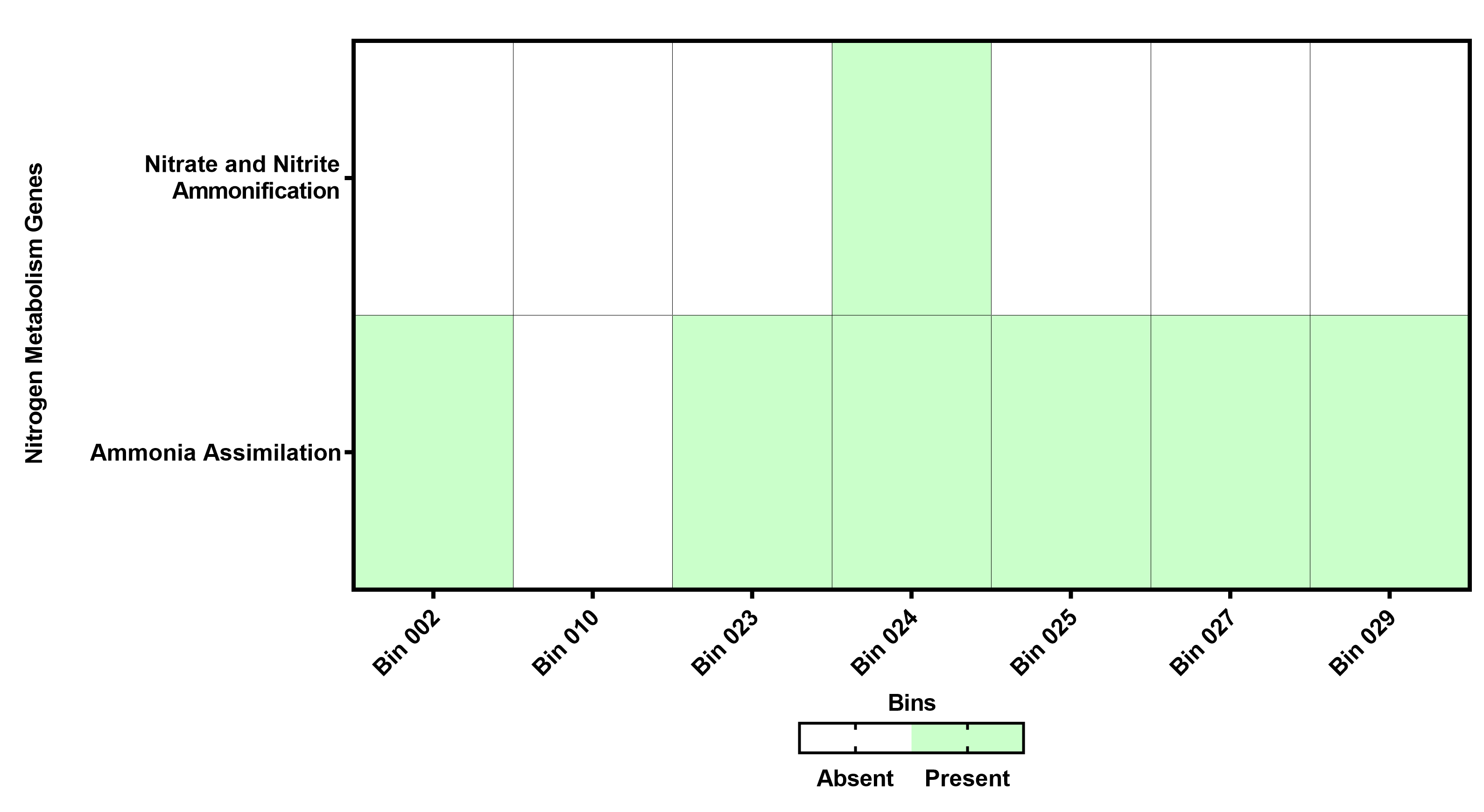


**Figure 1.** Nitrogen metabolism genes identified in the genomes using RAST through SEED viewer v.2.0.


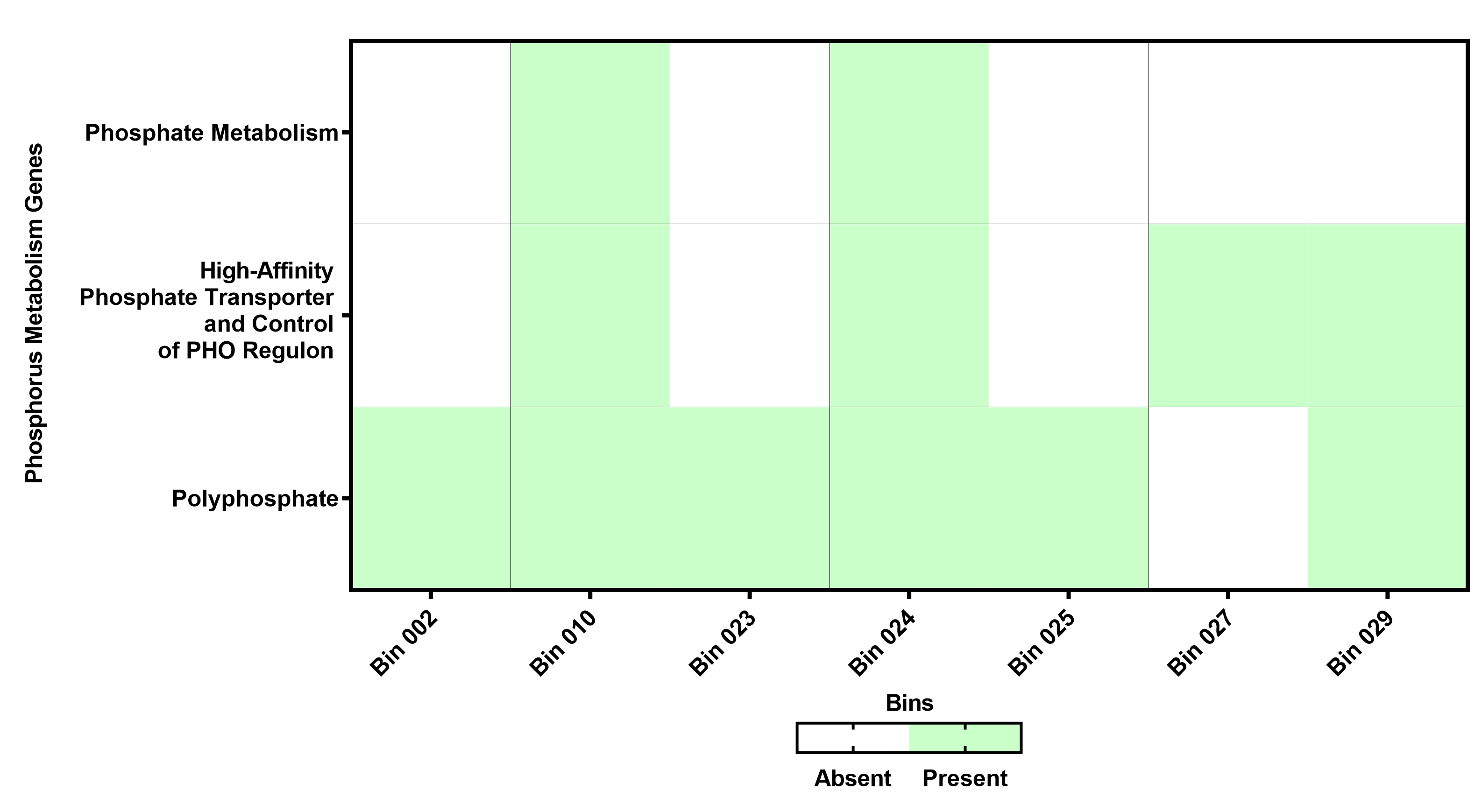


**Figure 2.** Phosphorus metabolism genes identified in the genomes using RAST through SEED viewer v.2.0.


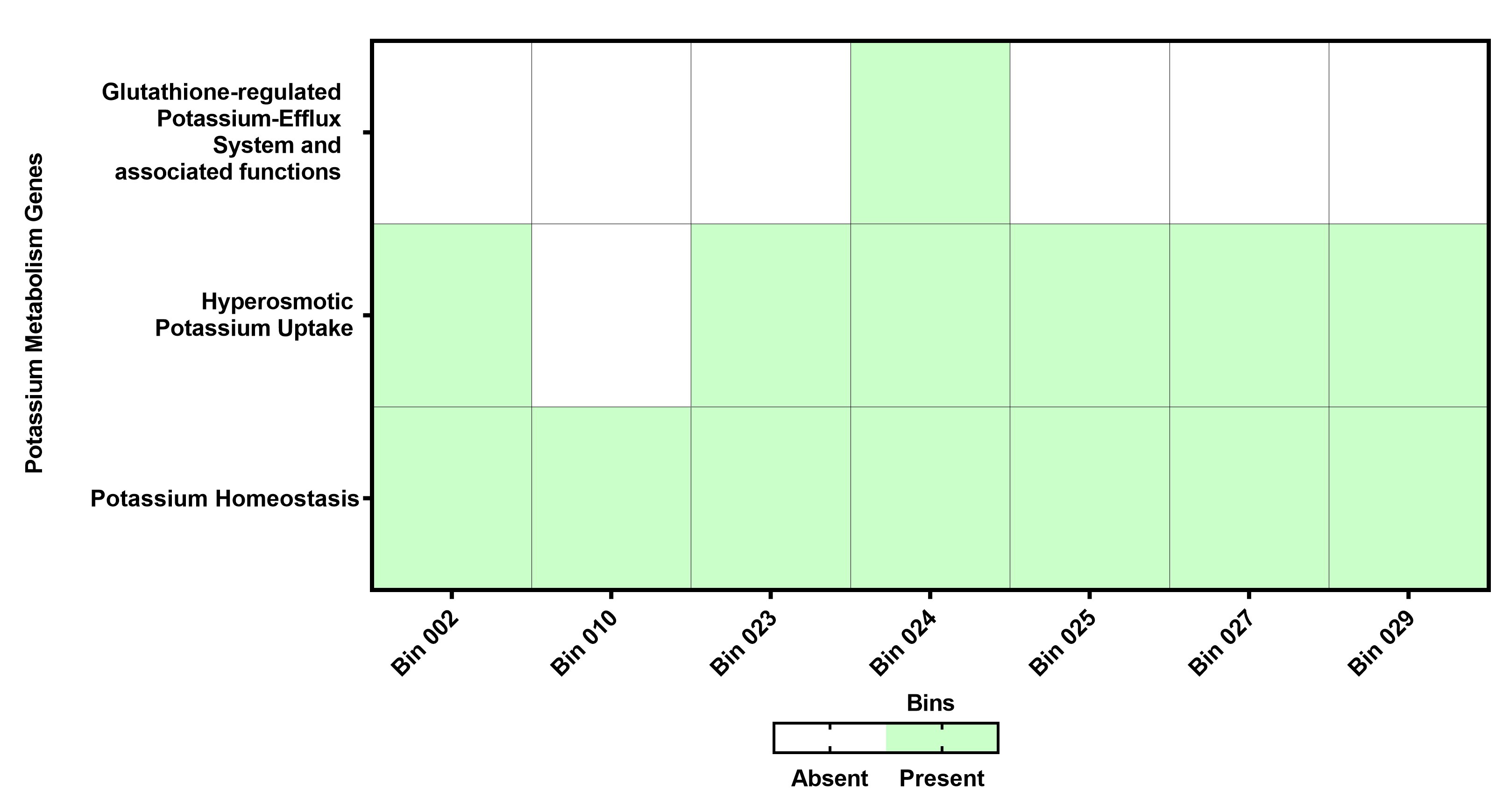


**Figure 3.** Potassium metabolism genes identified in the genomes using RAST through SEED viewer v.2.0.


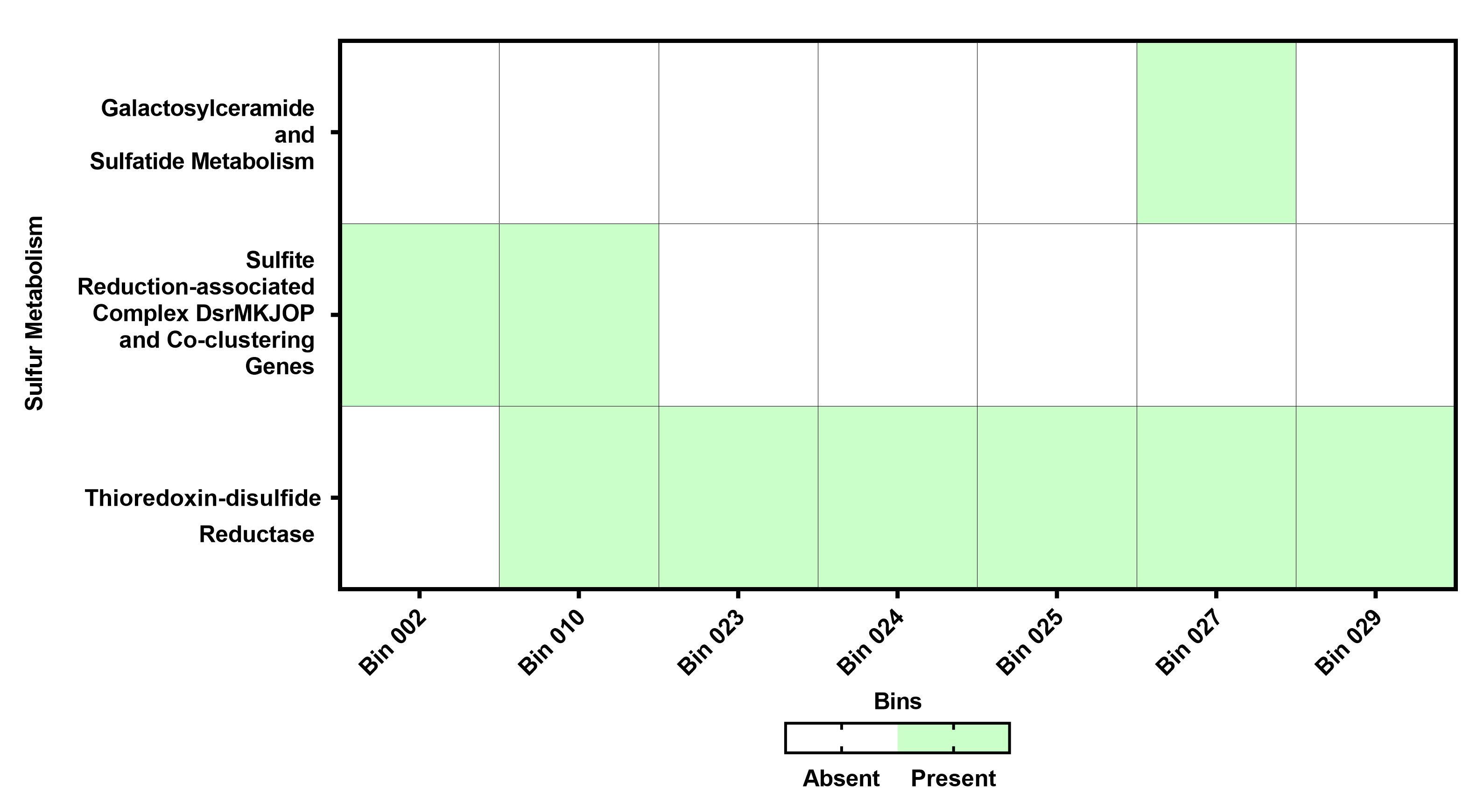


**Figure 4.** Sulfur metabolism genes identified in the genomes using RAST through SEED viewer v.2.0.


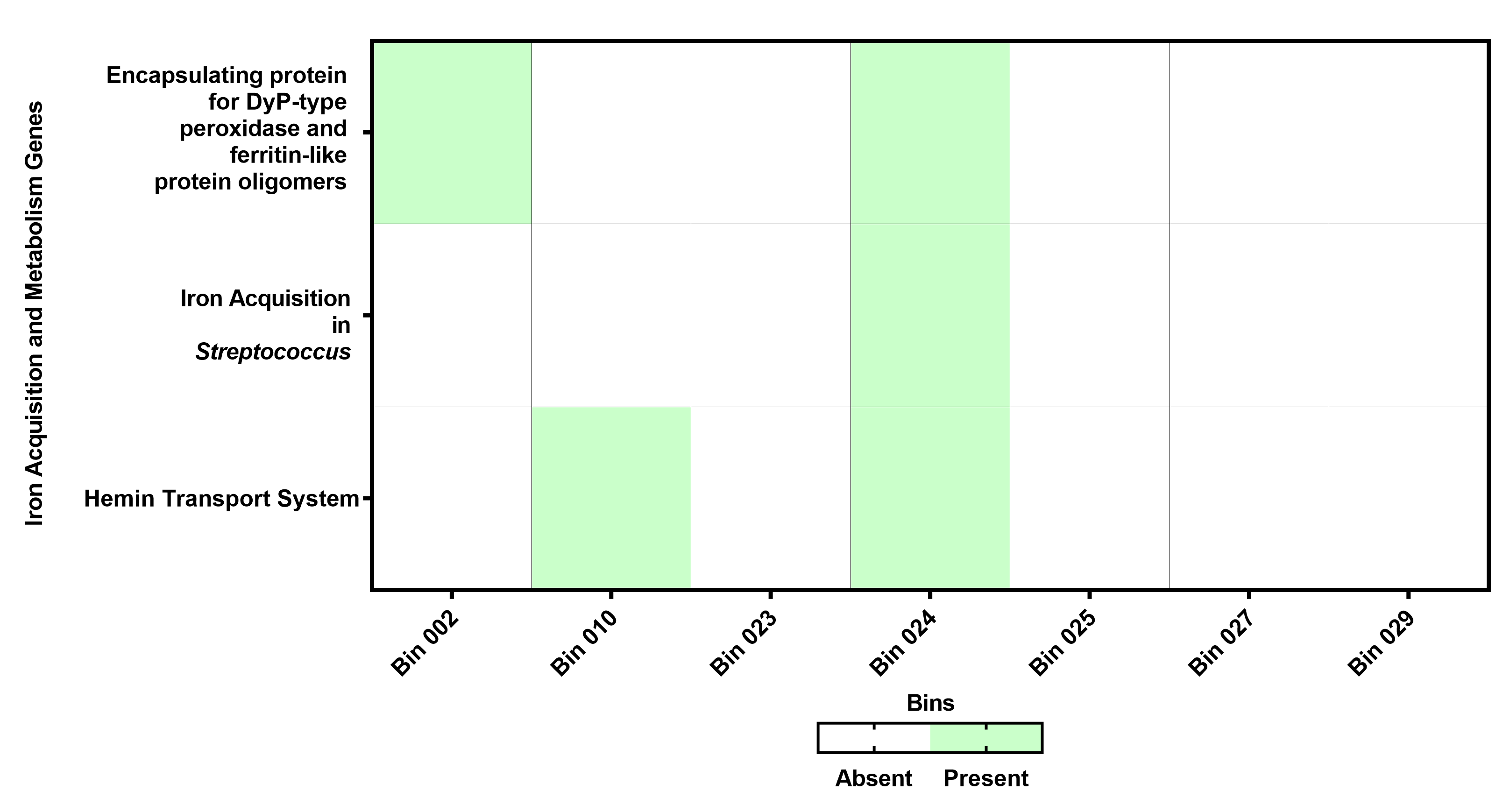


**Figure 5.** Iron acquisition and metabolism genes identified in the genomes using RAST through SEED viewer v.2.0.
