# Supplementary figures and images for: Exploring the taxonomical and functional profiles of marine microorganisms in Submarine Groundwater Discharge vent water from Mabini, Batangas, Philippines through metagenome-assembled genomes
Source: Front Genet. 2025 Feb 10;16:1522253. doi: 10.3389/fgene.2025.1522253 (PMC11868764; doi:10.3389/fgene.2025.1522253)

**Supplementary File 5**

**DRAM Results of the 7 MAGs**


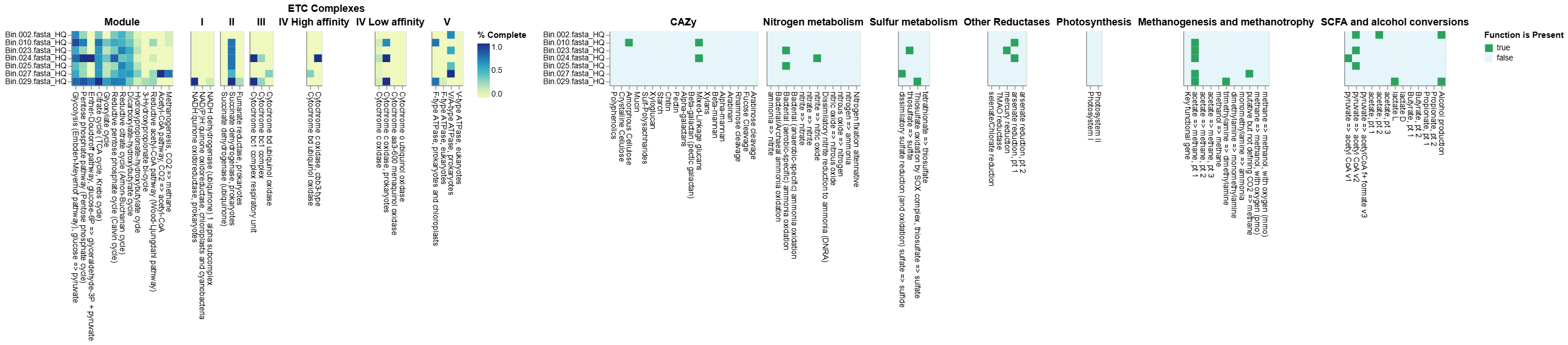


**Figure 1.** DRAM Annotations of the 2_1_SS_W3_F1 Bins.

Supplement: Supplementary file 5 [file Table4.docx]
