# Supplementary material for: Exploring the taxonomical and functional profiles of marine microorganisms in Submarine Groundwater Discharge vent water from Mabini, Batangas, Philippines through metagenome-assembled genomes
Source: Front Genet. 2025 Feb 10;16:1522253. doi: 10.3389/fgene.2025.1522253 (PMC11868764; doi:10.3389/fgene.2025.1522253)
Supplement: Supplementary file 6 [file Table6.docx]

**Supplementary File 6**

**antiSMASH Results of the Seven MAGs**

***Glaciecola***

**Figure 1.** Detected BGCs within the seven MAGs using antiSMASH v.7.0.

**Figure 2.** BGCs distribution within the seven MAGs predicted through antiSMASH v.7.0.
